# Metabolic and Microcirculatory Changes in Severe Renal Ischemia–Reperfusion and Ischemic Preconditioning in the Rat: Are They Detectable in the First Hour of Reperfusion?

**DOI:** 10.3390/life15040592

**Published:** 2025-04-03

**Authors:** David Martin Adorjan, Laszlo Adam Fazekas, Adam Varga, Adam Attila Matrai, Laszlo Bidiga, Tamas Lesznyak, Adam Deak, Katalin Peto, Norbert Nemeth

**Affiliations:** 1 Department of Operative Techniques and Surgical Research, Faculty of Medicine, University of Debrecen, Moricz Zsigmond str. 22, H-4032 Debrecen, Hungary; corvus280@mailbox.unideb.hu (D.M.A.); fazekas.laszlo@med.unideb.hu (L.A.F.); varga.adam@med.unideb.hu (A.V.); matrai.adam@med.unideb.hu (A.A.M.); lesznyak.tamas@med.unideb.hu (T.L.); deak.adam@med.unideb.hu (A.D.); kpeto@med.unideb.hu (K.P.); 2 Department of Pathology, Faculty of Medicine, University of Debrecen, Moricz Zsigmond str. 22, H-4032 Debrecen, Hungary

**Keywords:** ischemia–reperfusion, ischemic preconditioning, kidney, hemorheology, hemodynamics, microcirculation

## Abstract

Ischemia–reperfusion (I/R) strongly affects a graft’s function and survival and modulates microcirculatory and hemorheological parameters. However, the boundary between the reversibility and irreversibility of damage is unclear. This study compared the effects of renal I/R and ischemic preconditioning (IPC) to determine whether metabolic, microcirculatory, and micro-rheological changes are already detectable in the first hour of reperfusion. Wistar rats were divided into control (n = 6), I/R (n = 7) and IPC (n = 7) groups. In the ischemic groups the left kidney was subjected to 120 min of ischemia followed by 60 min of reperfusion. In the IPC group, a 3 × 5 min protocol was used prior to the manifest ischemia. Parenchymal microcirculation and renal artery blood flow were measured before ischemia (base) and during reperfusion (R-30, R-60). Hematological, micro-rheological parameters, electrolytes, and metabolites were tested at base and at R-60. Both ischemic groups showed micro-rheological impairment. An increase in potassium, lactate, and creatinine concentrations and a decrease in pH were observed. The blood flow of the IPC group deteriorated less, and microcirculation recordings indicated better values. The 120 min ischemia and the 60 min reperfusion resulted in micro-rheological and metabolic alterations, together with decreased renal blood flow and parenchymal microcirculation. Although the applied IPC protocol showed minor protective effects, its impact was limited in the first hour of reperfusion.

## 1. Introduction

End-stage renal failure is associated with acute or chronic kidney failure, and kidney transplantation is the only chance to improve quality of life and in many cases is the only option for survival [1,2,3,4]. The persistent shortage of organ donors has led to an intensified focus on maximizing the success of organ transplants [1,5,6,7,8,9,10,11]. A critical factor in transplant viability is the period when the circulation to the donor organ is halted, resulting in decreased oxygenation and the generation of reactive oxygen species (ROS) [6,8,9,10,11]. These ROS, along with oxidative stress, cause irreversible damage to tissues, especially during ischemia–reperfusion injury [4,5,12,13,14,15,16]. Among the renal structures, the proximal tubules and the thick ascending limb of the renal cortex are most vulnerable to hypoxia, given their high metabolic activity and oxygen demand [4,17,18]. The restoration of blood supply as soon as possible during transplantation is essential, as ischemic damage can cause organ dysfunction. The cessation of the blood supply of the organ to be transplanted is an inevitable part of the transplantation process [5,6,19]. During this period of warm ischemia, oxygenation is reduced due to vasoconstriction and vascular occlusion, and tubule cell damage is sustained [4,6]. Tubule cells lose their function, and the localization of adhesion molecules and membrane proteins is altered. The functional capacity of the transplanted organ will be a fraction of its original state. Microcirculatory changes of an acute and chronic nature play a major role in this [20,21,22,23]; however, the threshold and exact timing of the emergence of these changes, as well as their role in determining reversibility or irreversibility, remain unknown.

Recent advances in organ protection strategies during transplantation have highlighted the potential of conditioning protocols [24,25,26]. These protocols are designed to mitigate the damage associated with warm ischemia and reperfusion. Among these strategies, ischemic preconditioning (IPC) has emerged as a promising intervention [24,25,26,27]. IPC involves the deliberate and controlled interruption of blood flow to the organ in short repeated intervals prior to the sustained ischemic event [24]. This approach has been shown in the literature to have a positive impact on micro-rheological and microcirculatory parameters, thus providing protection against hypoxic injury during organ transplantation [28,29].

Hemorheology, the study of blood flow properties, is pivotal in understanding the pathophysiology of ischemia–reperfusion injury [21,30]. Blood viscosity, the deformability of red blood cells, and their aggregation tendencies are critical factors that influence microcirculation. Several studies showed that free radicals, metabolic, blood gas, and acid–base alterations, inflammatory processes, and mechanical trauma cause impairment in red blood cell deformability and aggregation [12,21,30,31,32,33,34,35]. These alterations result in a ‘vicious circle’ that reduces further tissue perfusion [33].

Previously, we studied the hemorheological and microcirculatory effects of renal ischemia–reperfusion of various durations and ischemic preconditioning protocols in follow-up studies, revealing a deterioration in parameters affecting tissue perfusion in the early postoperative hours and days [28,29,32,34,36]. However, it remains unclear whether these changes begin to manifest within the first hour of reperfusion.

We hypothesized that alterations in blood flow, microcirculatory, micro-rheological, acid–base, and metabolic parameters would already be detectable in the first hour of reperfusion and would progressively intensify over time.

This study investigated whether severe ischemia and the first hour of reperfusion, with or without ischemic preconditioning, induce detectable changes in hemorheological, hematological, microcirculatory, and metabolic parameters.

## 2. Materials and Methods

### 2.1. Experimental Animals and Operative Protocol

The study was conducted following the related European Union Directive (Directive 2010/63/EU) and National Regulations (The Hungarian Animal Protection Act, Law XXVIII/1998) and with the approval of the University of Debrecen Committee of Animal Welfare (reg. no.: 19/2022/UDCAW). Twenty male Wistar (Crl:WI) rats (age: 7–8 weeks; bodyweight: 213.83 ± 10.94 g; origin: Toxi-Coop Zrt., Budapest, Hungary) were involved in the study. The animals were kept in the Departments’ conventional animal facility (standard cages: Eurostandard IV, Tecniplast, Buguggiate, Italy; temperature: 22 ± 2 °C; humidity: 55% ± 10%; lighting: 12–12 h light/dark cycle) and had free access to water and standard rat food (SAFE^®^ D132 autoclavable complete universal vegetal diet for rats, mice, and hamsters, SAFE^®^ Complete Care Competence, Augy, France).

General anesthesia was provided using a combination of 100 mg/bwkg i.p. ketamine hydrochloride (CP ketamine hydrochloride 10%, Produlab Pharma BV, Raamsdonksveer, The Netherlands), 10 mg/bwkg i.p. xylazine hydrochloride (CP xylazine hydrochloride, 2%; Produlab Pharma BV, Raamsdonksveer, The Netherlands), and 0.05 mg/kg Atropin s.c. (Atropinum sulfuricum 0.1%, Egis Pharmaceuticals PLC, Budapest, Hungary) [37,38].

**Figure 1:**
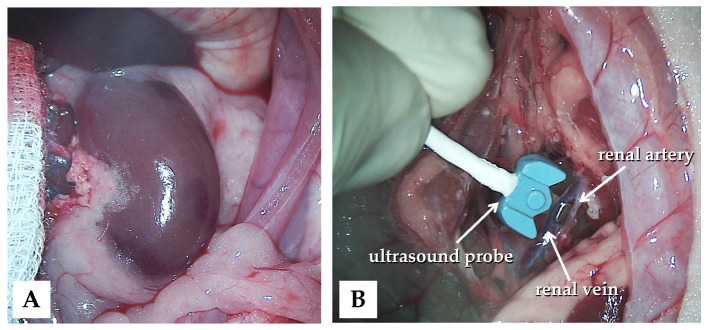
Intraoperative photos of the left kidney exploration (**A**) and the application of the blood flow measurement probe (**B**).

The animals were subjected to control (n = 6), ischemia–reperfusion (I/R, n = 7), or ischemic preconditioning (IPC, n = 7) groups. In all animals, a lateral tail vein was cannulated (26 G, Neoflon™ Pro IV Cannula; Becton, Dickinson and Company, Franklin Lakes, NJ, USA), and a median laparotomy and gentle preparation of the kidneys were performed (Figure 1A).

In the control group, no other intervention was made during the observation period. In the I/R and IPC groups, 120 min of ischemia was induced on the left kidneys using a microvascular clamp on both the renal artery and vein [28,29,39]. After releasing the clamps, 60 min of reperfusion was observed. In the IPC group prior to the 120 min ischemia, three cycles of 5 min of ischemia and 5 min of reperfusion were used for ischemic preconditioning.

Blood samples (0.5 mL/each; EDTA, Vacutainer^®^, Becton Dickinson GmbH, Franklin Lakes, NJ, USA) were collected from the cannulated lateral tail vein before surgery (base) and at the end of experimental procedures (60th minute of reperfusion, R-60). Renal vessel blood flow and microcirculatory measurements were performed before the manifest ischemia, and in the 30th and 60th minutes of the reperfusion. There was no mortality during the observation period. At the end of the observation period, the animals were euthanized intravenously (300 mg/kg, ketamine hydrochloride 10%; 30 mg/kg, xylazine hydrochloride 2%), and the kidneys were removed for histological analyses.

### 2.2. Laboratory Techniques

Blood gases (*p*O_2_ [mmHg], *p*CO_2_ [mmHg]), pH, electrolytes (Na^+^ [mmol/L], K^+^ [mmol/L], Ca^2+^ [mmol/L], Cl^−^ [mmol/L],), metabolites (lactate [mmol/L], and creatinine [µmol/L]) were determined by an EPOC Blood Analysis System (Siemens Healthineers AG, Erlangen, Germany).

For testing hematological parameters, a Sysmex K4500 microcell counter device (TOA Medical Electronics Corp., Ltd., Kobe, Japan) was used. In this paper, RBC count [10^12^/L], white blood cell count (WBC [10^9^/L]), platelet count (Plt [10^9^/L]), and hematocrit (Hct [%]) were analyzed.

A LoRRca Maxsis Osmoscan ektacytometer (RR Mechatronics International B.V., Zwaag, The Netherlands) was used to investigate red blood cell deformability [40]. The method is based on the analysis of laser-diffraction patterns as red blood cells are elongating by the applied shear stress (0.3–30 Pa). For the tests, 10 μL of blood was mixed with 2 mL of polyvinyl–pyrrolidone (PVP)–PBS solution (PVP: 360 kDa, Sigma-Aldrich Co., St. Louis, MO, USA; PVP-PBS solution viscosity: 33.3–33.6 mPas, osmolality: 290–310 mOsmol/kg, pH: 7.2–7.5). All measurements were carried out at 37 °C [41]. The elongation index (EI)–shear stress (SS) curves were analyzed further using the Lineweaver–Burk equation to determine the maximal elongation index (EI_max_) and the shear stress at half EI_max_ (SS_1/2_, [Pa]), as well as their ratio [42].

Red blood cell aggregation was tested by a Myrenne MA-1 erythrocyte aggregometer (Myrenne GmbH, Roetgen, Germany) [40]. In the samples (20 µL of blood), the aggregation index values were determined by a light transmission method for 5 or 10 s at stasis (shear rate: 0 s^−1^; M 5 sec and M 10 sec values) or at a low shear (shear rate: 3 s^−1^; M1 5 sec and M1 10 sec index values). The higher index values reflect enhanced red blood cell aggregation.

### 2.3. Blood Flow and Microcirculatory Investigations

Blood flow rate (mL/min) was measured using a Transonic flowmeter (Transonic Microcirculation Flowprobe; Transonic Systems Inc., Ithaca, NY, USA), with the ultrasound probe positioned around the aorta and renal arteries and veins bilaterally (Figure 1B).

Microcirculatory investigations of the kidneys were performed using a Cytocam-IDF video microscope (Braedius Medical, Huizen, The Netherlands) [43,44]. The device uses green laser light at 540 nm to capture high-resolution images of microvessels by epi-illuminating superficial tissue layers (2–3 mm deep). Offline analysis (CytoCamTools V3 Bedside Manager, Braedius Medical, Huizen, The Netherlands) of the recordings was completed to determine perfused vessel density (PVD, [mm/mm^2^]), the proportion of perfused vessels (PPV, [%]), and the microvascular flow index (MFI, [au]) [43,44].

### 2.4. Histological Analysis

Kidneys were fixed in a 10% formaldehyde solution and embedded in paraffin. On serial sections, hematoxylin–eosin staining was applied (H&E, Sigma-Aldrich, St. Louis, MO, USA). After digitalization (VENTANA DP200, Hoffmann-La Roche, Roche Holding AG, Basel, Switzerland), general histomorphological analysis was conducted by a pathologist. Although a 60 min period after ischemia is too short to realistically show apoptotic processes, intact, pyknotic, and hydropic cells were also counted (open source software: QuPath 0.4.4; ImageJ 1.40).

### 2.5. Statistical Analysis

The required sample size (number of animals per group) was determined using Mead’s resource equation method. SigmaStat Software 3.1.1.0. (Systat Software Inc., San Jose, CA, USA) was used for statistical analyses.

Data are presented as means ± standard deviation (S.D.). According to the results of the normality test, for inter-group comparison, the *t*-test or the Mann–Whitney rank-sum test was used, and for intra-group comparison, a one-way ANOVA or Kruskal–Wallis’s test was used.

A *p*-value of <0.05 was considered statistically significant.

## 3. Results

### 3.1. Blood Gases, pH, Electrolytes, and Metabolites

In the I/R and IPC groups, *p*O_2_ values significantly decreased by the 60th minute of the reperfusion, while an increase in *p*CO_2_ was observed. A significant decrease in pH was also observed in both ischemic groups (*p* < 0.001 vs. base and control in both groups), together with a rise in lactate concentration. In the I/R group, the lactate concentration increase was significant compared to its base values (*p* = 0.043) and to the control group at the same time point (*p* = 0.021). In the IPC group, the rise was significant only compared to the base (*p* = 0.043), while versus the control group, the *p* value was 0.077. Notably, potassium and creatinine concentrations rose more prominently in the IPC group (Table 1).

### 3.2. Hematological Parameters

A reduction in white blood cell count was observed in both ischemic groups by the end of the reperfusion period that was significant in the I/R group versus the control group (*p* < 0.001). Hemoconcentration was evidenced by an increase in red blood cell count (in I/R group *p* < 0.001 vs. its base) and hematocrit (in I/R group *p* < 0.001 vs. base and vs. control group; in IPC group *p* < 0.001 vs. its base). Platelet count significantly increased both in the I/R and IPC groups compared to the control group by the 60th minute of the reperfusion (*p* < 0.001 in each) (Table 2).

### 3.3. Micro-Rheological Parameters

**Figure 2:**
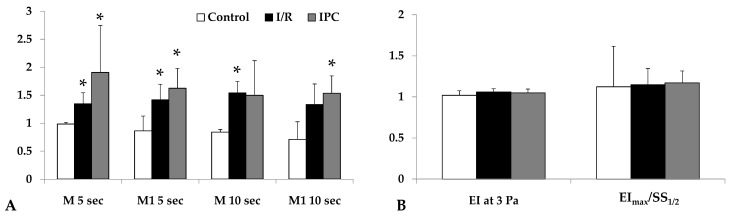
Alterations in the red blood cell aggregation index (relative to their base values) tested at the 5th and 10th second of the aggregation process under stasis (M 5 sec, M 10 sec) and at 3^−1^ shear rate (M1 5 sec, M1 10 sec) values (**A**); red blood cell deformability (elongation index at 3 Pa, ratio of EI_max_ and SS_1/2_) parameters (**B**) in control, I/R, and IPC groups, with baseline and values at the 60th minute of the reperfusion (R-60). Mean ± S.D.; * *p* < 0.05 vs. control group.

Red blood cell aggregation, measured by light intensity during aggregation assays, increased significantly by the end of reperfusion in both I/R and IPC groups compared to the control (in I/R group: M 5 sec: *p* = 0.029, M1 5 sec: *p* = 0.012, M 10 sec: *p* < 0.001, M1 10 sec: *p* = 0.036; in IPC group: M 5 sec: *p* = 0.032, M1 5 sec: *p* = 0.007, M 10 sec: n.s., M1 10 sec: *p* = 0.024). The IPC group displayed higher aggregation index values. Red blood cell deformability did not show important changes (Figure 2).

### 3.4. Blood Flow Rates

**Figure 3:**
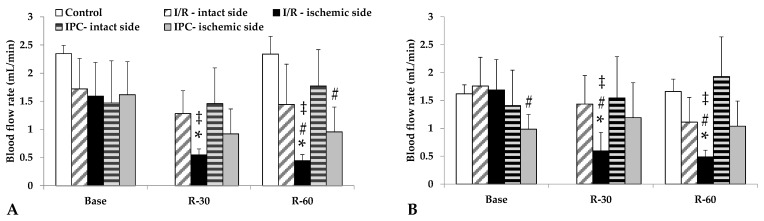
Changes in blood flow rates of renal artery (**A**) and renal vein (**B**) in the control group and of intact and ischemic kidneys of the I/R and IPC groups. Values of baseline (as base, before ischemia) and at the 30th and 60th minutes of the reperfusion (R-30, R-60). Mean ± S.D.; * *p* < 0.05 vs. base; # vs. control group at the same time point; ‡ vs. intact side vessel.

Blood flow in the renal arteries, normally lower than in the aorta, deteriorated further in the ischemic kidneys, a condition that had not normalized by 60 min of reperfusion. In the I/R group, the differences were significant compared to its base values, as well as versus the intact side and control group values at the same time point (*p* < 0.001, each). The IPC group demonstrated a less severe decrease in flow; however, the ischemic side values were lower than those of the control group (Table 3, Figure 3).

### 3.5. Microcirculatory Parameters

The perfused vessel density, the proportion of perfused vessels, and the microvascular flow index did not show significant changes. However, values of the I/R and IPC groups remained lower at the end of the observation period compared to the control. The IPC group expressed a more visible decrease in microcirculatory values due to the applied short cycles of ischemia. The magnitude of restoration was higher in the IPC group (Table 4).

### 3.6. Histology

**Figure 4:**
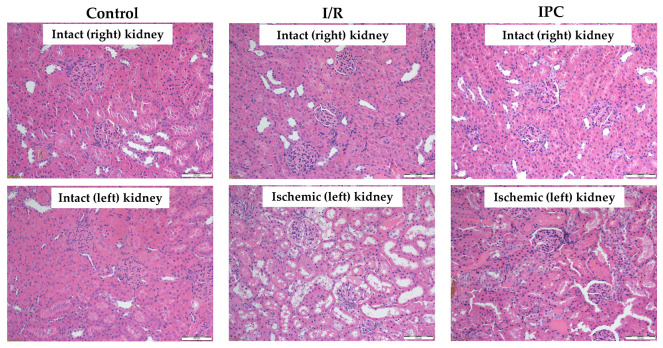
Representative histological images of kidney samples of control (intact right and left), I/R (intact right, ischemic left), and IPC groups (intact right, ischemic left). Hematoxylin–eosin stain, original magnification: 200×, scale bar: 100 μm.

**Figure 5:**
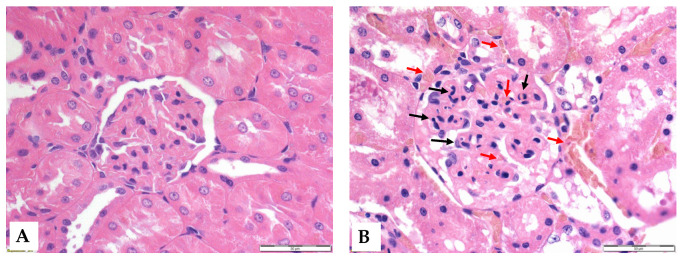
Representative histological images of the glomeruli of control (**A**) and I/R (**B**) groups. Red arrows: red blood cell stasis; black arrows: endocapillary exudates. Hematoxylin–eosin stain, original magnification: 600×, scale bar: 50 μm.

Histological analysis revealed that ischemia caused significant morphological changes, with a marked deterioration of the tubular epithelium in the ischemic and preconditioned ischemic kidneys compared to intact control kidneys. The observed alterations included red blood cell trapping in the glomerular and peritubular capillaries, brush border fragmentation, epithelial cell detachment, absence of nuclear staining, epithelial flattening, reduction in organelle content, hydropic degeneration, and tubular dilation. There was glomerular endocapillary hypercellularity, characterized by an increased number of inflammatory cells, including neutrophils (Figure 4 and Figure 5).

A higher proportion of pyknotic cells was seen in the ischemic and preconditioned ischemic kidneys compared to intact ones (Table 5).

## 4. Discussion

Kidney ischemia and ischemia–reperfusion injury are still widely investigated issues because of their importance in clinical practice, especially related to urological operations and kidney transplantation procedures [1,4,9,13,18]. The pathophysiology of ischemic renal injury is complex, in which tissue perfusion-related factors play an important role in the aspect of various damage-reducing or damage-preventing possibilities [4,8,10,12,14].

Murry and his colleagues first published their article on the so-called ischemic preconditioning process in 1986, by which they observed a reducing effect on manifest ischemia–reperfusion injury [24]. They observed that short repetitive ischemic stress induces certain cellular and metabolic protective mechanisms, whereby cells are more likely to survive a prolonged ischemic state without apoptosis or necrotic cell death. Subsequently, other research groups have described several protocols in the literature (preconditioning, postconditioning, remote organ preconditioning) [24,26,27,30,45,46,47,48]. Preconditioning involves exposing the organ to ischemia at repetitive short intervals during sustained ischemia, thereby achieving the activation of cell and tissue defense mechanisms. In this way, it is possible to mitigate cell damage that occurs during a prolonged period of ischemia. Its clinical application is not yet widespread, but there are several case reports of its use in reducing perioperative ischemic damage [4,6,16,45].

Histopathological studies have demonstrated that the long-term viability of renal tissue cells is influenced by ischemia duration, but little is known about when the earliest changes become detectable, particularly in the first hour of reperfusion. With the loss of oxygen and nutrient supply, followed by oxidative stress after reperfusion, the state of the cells deteriorates steadily [17]. The early milder damage results in so-called hydropic degeneration. In this case, water accumulates in the cytoplasm of the cells, the cells swell, and morphological changes develop. These hydropic abnormalities are mostly reversible for a short time after the ischemic state. After the restoration of normal circulation and improvement of oxygenation, the cells may regain their original morphological and functional state [14,17]. However, 60 min after ischemia is too short to present such alterations, as more time is needed for apoptotic processes.

If ischemia persists, hydropic degeneration is followed by severe damage to the cell membrane and internal cellular structures. At this stage, irreversible changes such as shrinkage (pyknosis), fragmentation (karyorrhexis), and dissolution (karyolysis) of the nucleus appear. If the endogenous reparative mechanisms of the cells fail, apoptosis may also occur. Global damage to the organ may result in the detachment of tubular epithelial cells from the basement membrane, and inflammatory processes may exacerbate tissue destruction. In such cases, the process is irreversible, and the renal tissue suffers permanent damage [8]. Our data showed a significant difference in the proportion of pyknotic cells. In both experimental groups, the pyknosis rate was significantly higher than in control kidneys, but there was no significant difference between the two groups. This indicates that the preconditioning protocol used was not sufficient to mitigate the tissue damage caused by this degree of ischemia in the first hour of the reperfusion.

Regarding hematological parameters, a significant decrease in leukocyte count was observed in both groups at the end of reperfusion. This difference in numbers was probably caused by leukocytes precipitating along the locally damaged vascular network during the ischemic phase. Thus, the leukocyte count in the circulating blood is temporarily lower, while the true leukocyte count is not necessarily reduced; rather, this is a distributional difference [49]. A reduced circulating leukocyte number is supposed to be due to sequestration in the damaged kidney (Figure 5). In contrast, a significant increase in RBC count was observed, largely explained by the phenomenon of hemoconcentration. Following ischemia, fluid redistribution occurs, so plasma volume may temporarily decrease, resulting in an increase in the relative proportion of blood cells [21,30,50].

The principal findings were the differences in red blood cell aggregation between control and ischemic groups (Figure 2) and the lower arterial and venous renal blood flow values of the I/R group in comparison to the other groups at the 30th and 60th minutes of the reperfusion. In both groups, the red blood cell aggregation parameters increased, but these showed significant changes only in the IPC group. For the red blood cell deformability of both ischemic groups, it showed a slight deterioration by the 60th minute of reperfusion; however, it was not significant. Micro-rheological parameters of the erythrocytes can be altered by free radical damage, blood gases, acid–base, and metabolic alterations [21,30,33]. A significant decrease in blood pH was observed in both groups. It is a well-known phenomenon during ischemia–reperfusion, explained by the increase in lactate levels also observed in our measurements [51]. In addition, the rise in potassium ion concentration can be explained by the metabolic acidosis that occurs during ischemia, leading to hyperkaliemia as potassium is transferred from the intracellular space to the extracellular compartment. On the other hand, the rise in creatinine concentration was triggered by the persistent ischemia, as the glomerular filtration rate (GFR) also decreased with the cessation of arterial blood flow. Since creatinine is almost exclusively excreted via the kidneys, a decrease in the GFR leads to an increase in creatinine concentration [4].

Measurements of blood flow velocity in the blood vessels showed that renal blood flow in the IPC group decreased to a lesser extent after permanent ischemia and then showed no significant difference in the reperfusion phase compared to the intact state. This suggests that the IPC protocol maintained the renal blood flow rate within the normal flow range, even under significant stress. The post-experimental analysis of the intraoperative microcirculation images shows that blood in the vessels stagnates, with an increased aggregation of red blood cells. By the 60th minute of reperfusion, the microcirculatory pattern began to resemble the control, but parenchymal edema persisted.

After numerical processing of the recordings, a significant increase in the proportion of perfused capillaries was seen. This is presumably due to the strong vasodilatation induced by the release of large amounts of nitric oxide (NO) following prolonged oxygen deprivation [52]. This may result in more blood moving to the hypoxic area, which may paradoxically worsen the reperfusion injury. In the pre-conditioned group, this capillary dilatation was not so pronounced. This is possible due to the protective mechanisms triggered by the IPC protocol used to balance the circulatory and oxidative processes. NO causes strong vasodilatation, which worsens the reperfusion injury in the affected area. However, this phenomenon was less pronounced in the IPC group [52].

Limitations of the study include the potential inter-species differences, comparability of experimental models versus clinical cases [33,53,54,55], the relatively low case number, the short observation period, and difficulties in the clear diversification of the effects of the repeated short-time ischemic cycles and the ongoing manifest ischemia. The volume and repeatability of blood samplings were limited, related to body weight, circulating blood volume, laboratory animal science principles, and the required blood sample volume for laboratory measurements. In this experiment, blood samples of 0.5 mL were taken twice within 3 h. As we have previously studied the consequences of renal ischemia [28,32], in our current study, we focused more on reperfusion injury. In previous studies, we could better differentiate the ischemic- and the reperfusion-related alterations [28,29,32]; however, the multiple blood sampling was considered a strong limitation and an influencing factor. However, in a large animal model, this would have been better investigated [36]. More blood samplings would have affected the hemodynamics and blood parameters, which would have made it difficult to objectively evaluate the reperfusion results. However, the initial processes after ischemia have not been revealed completely yet; therefore, we assume that these findings showing acute blood flow, microcirculatory, micro-rheological, acid–base, and metabolic effects in parallel may provide useful information for further studies to optimize preconditioning protocols.

## 5. Conclusions

Severe warm ischemia and reperfusion caused detectable changes in hematological, hemorheological, and metabolic parameters, as well as in the renal vessel blood flow and parenchymal microcirculation, which were slightly mitigated by the applied IPC protocol. The applied ischemic preconditioning protocol showed some protective effects on renal blood flow and microcirculation; however, in the first hour of reperfusion, no clear metabolic or micro-rheological changes were observed that would explain the well-documented long-term effects of ischemic preconditioning.

## Figures and Tables

**Table 1:** Changes in pH, *p*O_2_, *p*CO_2_ values, potassium (K^+^), lactate, and creatinine concentrations in the control, I/R, and IPC groups, with baseline and values at the 60th minute of the reperfusion (R-60).

Variable	Sample	Control	I/R	IPC
pH	base	7.41 ± 0.06	7.45 ± 0.06	7.41 ± 0.07
	R-60	7.36 ± 0.01	7.04 ± 0.09 *#	7.1 ± 0.08 *#
*p*O_2_ [mmHg]	base	64.68 ± 6.13	67.2 ± 7.95	62.77 ± 16.55
	R-60	65.63 ± 1.33	40.51 ± 13.28 *#	39.67 ± 6.93 *#
*p*CO_2_ [mmHg]	base	39.7 ± 4.08	33.05 ± 6.9	37.51 ± 8.65
	R-60	45.2 ± 1.83	70.6 ± 13.68 *#	55.87 ± 13.04 *#
K^+^ [mmol/L]	base	3.63 ± 0.64	3.8 ± 0.44	3.41 ± 0.68
	R-60	3.76 ± 0.51	4.54 ± 1.07	4.91 ± 0.05 *#
lactate [mmol/L]	base	1.81 ± 0.64	1.91 ± 0.74	1.96 ± 1.06
	R-60	1.73 ± 0.74	3.86 ± 1.76 *#	3.41 ± 1.78 *
creatinine [μmol/L]	base	0.35 ± 0.03	<0.3	<0.3
	R-60	0.41 ± 0.06	0.71 ± 0.28	0.76 ± 0.29

Mean ± S.D.; * *p* < 0.05 vs. base; # vs. control group at the same time point. The creatinine test in the device does not detect exact values under 0.3 μmol/L.

**Table 2:** Changes in selected hematological parameters in control, I/R and IPC groups, with baseline and values at the 60th minute of the reperfusion (R-60).

Variable	Sample	Control	I/R	IPC
WBC [10^9^/L]	base	8.92 ± 1.64	7.31 ± 2.67	9.8 ± 5.84
	R-60	7.2 ± 0.62	5.51 ± 1.37 #	6.17 ± 2.18
RBC [10^12^/L]	base	6.58 ± 0.35	6.95 ± 0.87	6.35 ± 0.9
	R-60	7.39 ± 0.24	8.47 ± 0.61 #	8.07 ± 1.75
Hct [%]	base	40.83 ± 4.82	39.99 ± 10.69	38.95 ± 4.33
	R-60	43.82 ± 0.52	55.61 ± 3.13 *#	52.62 ± 9.8 *
Plt [10^9^/L]	base	750.8 ± 96.8	849.4 ± 345.9	803.4 ± 197.4
	R-60	426 ± 76.5	1014 ± 99.1 #	989.5 ± 186 #

Mean ± S.D.; * *p* < 0.05 vs. base; # vs. control group at the same time point. WBC: white blood cell count; RBC: red blood cell count; Hct: hematocrit; Plt: platelet count.

**Table 3:** Blood flow rate [mL/min] in the abdominal aorta of control, I/R, and IPC groups, with baseline and values at the 60th minute of the reperfusion (R-60).

Variable	Sample	Control	I/R	IPC
blood flow rate [mL/min]	base	4.26 ± 0.5	3.94 ± 0.62	4.66 ± 1.05
	R-60	4.04 ± 0.39	3.81 ± 0.6	4.09 ± 1.01

Mean ± S.D.

**Table 4:** IDF videomicroscope parameters in the control group and of intact and ischemic kidneys of the I/R and IPC groups. Values of baseline (as base, before ischemia) and at the 60th minute of the reperfusion (R-60).

Variable	Sample	Control Group	I/R Group		IPC Group	
			Intact Side	Ischemic Side	Intact Side	Ischemic Side
PVD [mm/mm^2^]	base	4.77 ± 0.27	4.38 ± 2.05	4.99 ± 1.79	4.26 ± 1.2	3.36 ± 2.99
	R-60		4.18 ± 4.11	4.84 ± 1.78	2.98 ± 0.74	3.96 ± 1.53
PPV [%]	base	27.29 ± 2.78	25.58 ± 9.71	25.56 ± 12.64	26.48 ± 12.22	22.45 ± 19.08
	R-60		22.15 ± 16.47	29.53 ± 11.68	19.03 ± 6.76	28.08 ± 13.47
MFI [au]	base	2.85 ± 0.36	2.83 ± 0.41	2.83 ± 0.4	2.8 ± 0.42	2.9 ± 0.41
	R-60		2.72 ± 0.46	2.54 ± 0.52	2.7 ± 0.48	2.63 ± 0.51

Mean ± S.D. PVD: perfused vessel density; PPV: proportion of perfused vessels; MFI: microvascular flow index.

**Table 5:** Ratio of intact, pyknotic, and hydropic parenchymal cell counts in the control group and in intact and ischemic kidneys of the I/R and IPC groups.

Ratio of Cells/View	Lateralization	Control	I/R	IPC
Intact cells [%]	intact side	67.91 ± 4.76	79.76 ± 14.82	69.30 ± 5.11
	ischemic side	n.a.	27.54 ± 13.16 *#	17.36 ± 4.42 *#
Pyknotic cells [%]	intact side	23.34 ± 3.67	10.86 ± 5.94 #	16.86 ± 4.41 #
	ischemic side	n.a.	20.96 ± 8.07	26.36 ± 12.22
Hydropic cells [%]	intact side	11.04 ± 4.53	9.37 ± 9.52	20.68 ± 4.99 #
	ischemic side	n.a.	51.48 ± 11.12 *#	56.27 ± 9.38 *#

Means ± S.D.; * *p* < 0.05 vs. intact kidney; # vs. control group.

## Data Availability

The data presented in this study are available upon request from the corresponding author. The data are not publicly available due to ethical constraints.
